# The role of the gut microbiota and bile acids in heart failure: A review

**DOI:** 10.1097/MD.0000000000035795

**Published:** 2023-11-10

**Authors:** Min Shi, Jiaming Wei, Hui Yuan, Ya Li, Zhihua Guo

**Affiliations:** a Hunan University of Chinese Medicine, Changsha, China; b Hunan Key Laboratory of Colleges and Universities of Intelligent Traditional Chinese Medicine Diagnosis and Preventive Treatment of Chronic Diseases of Hunan, Changsha, China.

**Keywords:** bile acids, gut microbiota, heart failure, metabolites

## Abstract

Heart failure (HF) is the terminal manifestation of various cardiovascular diseases. Recently, accumulating evidence has demonstrated that gut microbiota are involved in the development of various cardiovascular diseases. Gut microbiota and their metabolites might play a pivotal role in the development of HF. However, previous studies have rarely described the complex role of gut microbiota and their metabolites in HF. In this review, we mainly discussed bile acids (BAs), the metabolites of gut microbiota. We explained the mechanisms by which BAs are involved in the pathogenesis of HF. We also discussed the use of gut microbiota and BAs for treating HF in Chinese medicine, highlighting the advantages of Chinese medicine in treating HF.

## 1. Introduction

Heart failure (HF) is the final manifestation of various cardiovascular diseases, such as valvular heart disease, cardiomyopathy, myocarditis, and myocardial infarction. HF is characterized by impaired ejection function or ventricular filling due to abnormal structural and functional alterations.^[1]^ Mortality and morbidity associated with HF remain high, and despite new drugs and treatment strategies in recent years, HF remains a leading cause of death worldwide.^[2–5]^ The current treatment and research mainly focus on angiotensin-converting enzyme inhibitors, angiotensin II receptor blockers, and statins.^[6]^ It is of great significance to clarify the pathogenesis of HF and further explore new therapeutic targets to reduce the economic burden of HF and improve patients quality of life.

Many studies have shown that gut microbiota are directly or indirectly involved in the development of various cardiovascular diseases.^[7]^ Due to reduced cardiac output and altered systemic circulation in HF, there is inadequate gut perfusion, which causes mucosal ischemia, intestinal edema, and impaired function of the gut barrier. In addition to increased gut permeability and altering the gut dysbiosis, the compromised gut barrier may allow microbial penetration into the systemic circulation and aggravate the chronic inflammation in patients with HF.

The "Gut hypothesis" focuses on gut bacterial translocation, enhanced inflammatory response, and oxidative stress,^[8]^ but not on the relationship between microbial metabolites and HF. Bile acids (BAs) are produced by cholesterol decomposition in the liver. Recent studies have found that BAs production is affected by the composition and function of intestinal flora, and abnormal metabolism of BAs can cause a severe inflammatory response and endothelial dysfunction, thereby increasing the risk of HF.^[7]^ Therefore, in this review, we discussed how gut microbiota and their metabolites, BAs, participate in HF. Herein, we reviewed the underlying mechanisms and summarized the current approaches and progress of Chinese medicine.

## 2. Gut microbiota dysbiosis in HF

The gut is the biggest microeco system in humans, gut microbiota are form a complex biological community made of up to 100 trillion microorganisms.^[9–10]^ As a "metabolite filter", its function is strongly associated with daily activities.^[11]^ Therefore, gut microbiota must be in dynamic equilibrium to protect the mucosal barrier, prevent pathogen colonization, and maintain human health. If the gut microbiome is unbalanced, pathogenic microorganisms may outnumber nonpathogenic microorganisms, which may affect the progression and pathogenesis of various chronic diseases, particularly HF.^[12]^

HF and gut microbiota have been linked by many studies. The imbalance of gut microbiota refers to abnormal intestinal microecology, which mainly manifests with altered quantity, proportion, and localization of species. Localization metastasis refers to the translocation of intestinal flora and their products such as endotoxin. HF development and progression can damage the intestinal mucosa, leading to gut dysbiosis and systemic inflammatory response. By inducing the inflammatory response and unfavorable reactions such as cachexia in the late stages of HF, the dysfunctional microbiota further exacerbate HF.^[8]^

**Figure 1.**
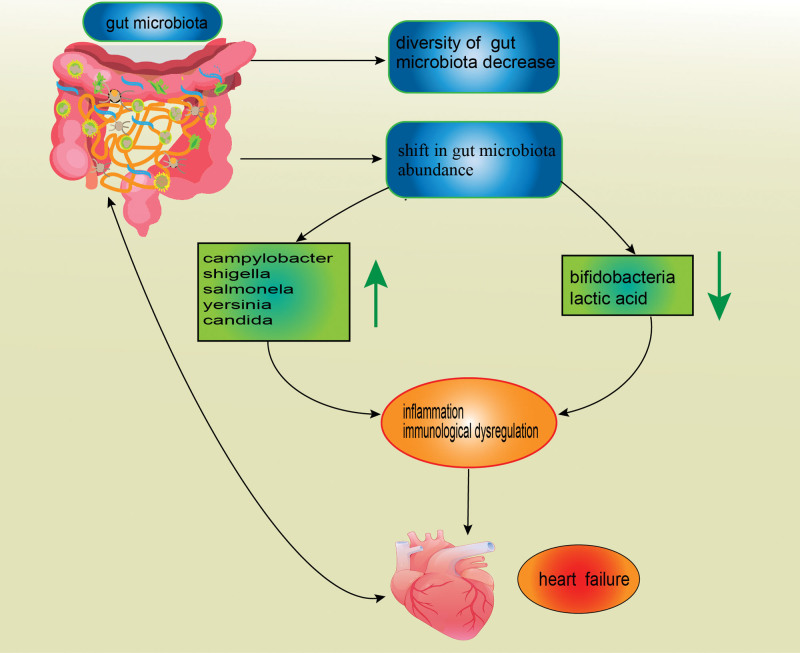
Gut dysbiosis, including decreased abundance of protective bacteria and increased abundance of harmful bacteria, causes chronic inflammation, autoimmune diseases, and heart failure (HF).

Patients with HF have gut dysbiosis, characterized by altered gut microbial composition and decreased diversity of gut microbiota.^[13]^ Decreased abundance of protective bacteria like bifidobacteria and lactic acid bacteria, and increased abundance of pathogenic bacteria such as campylobacter, shigella, salmonella, yersinia, and candida have been detected in the feces of patients with HF.^[13]^ The composition of gut microbiota in patients with HF significantly differs from that in normal subjects, although it is very similar among different subgroups of HF.^[14]^ A study^[15]^ indicated that patients with HF have a higher abundance of Synergistetes, Enterococcus and Lactobacillus and a lower abundance of Butyricicoccus, Sutterella, Lachnospira, and Ruminiclostridium. Additionally, it was unfolded that some pathogenic bacteria, including Shigella, Campylobacter, and Candida, are associated with the severity of HF, which can serve as prognostic markers in HF.^[16]^ It has also been shown that microbial dysbiosis can lead to immune dysregulation, chronic inflammation, and deterioration of HF with decreased ejection fraction.^[17]^ Figure 1 displays the relationship between gut microbiota and HF.

## 3. Gut microbiota and BAs

Gut microbiota and BAs have bidirectional interactions. BAs can alter the composition and function of gut microbiota; in return, gut microbiota modulate BAs metabolism. BAs also regulate innate immune system and bacterial defense, thereby influencing the composition of gut microbiota. Gut microbiota can also regulate BAs synthesis and their signaling pathways through BAs hydrolase. When BAs are released into the gastrointestinal tract, the gut microbiota can further metabolize BAs. This process includes debinding, oxidation, differential isomerization, 7-dehydroxylation, rebinding, and other reactions.^[18]^

### 3.1. Debinding

When the combined BAs are released into the intestine, hydrolysis of BAs by intestinal flora is the first step of their metabolism. Bacterial bile salt hydrolas is responsible for hydrolysis. Several species of bacteria, such as Lactobacillus, Clostridium, Enterococcus, and Bifidobacterium, can produce BA hydrolase.

### 3.2. 7-Dehydroxylation reaction

In addition to hydrolytically binding BAs, 7-dehydroxylation of BAs is one of the most important pathways of metabolic transformation. Clostridium and eubacteria are responsible for 7-dehydroxylation through a series of catalytic reactions, which involves a cascade of enzymatic activities mediated by the Bai gene cluster. The Bai gene cluster consists of 8 genes, with 7 enzyme-encoding genes and 1 transporter protein-encoding gene. After dehydroxylation, cholic acid (CA) is converted into deoxycholic acid (DCA) and chenodeoxycholic acid (CDCA) is converted into lithocholic acid (LCA).

### 3.3. Oxidation and differential isomerization

Differential isomerization of BAs promotes the enrichment of secondary BA species. Differential isomerism of BAs consists of 2 steps and can occur on the hydroxyl groups at the 3, 7, and 12 positions. BAs can be isomerized in 2 steps on the hydroxyl group at positions 3, 7, and 12. In the position 7 of CDCA, the hydroxy group at position 7 of CDCA is oxidized and dehydrogenby position-specific hydroxysteroid dehydrogenase to form 7-oxoLCA, which is the oxidation step. Then, another position-specific hydroxysteroid dehydrogenase is reduced to form ursodeoxycholic acid. Two steps are combined to convert the 7-αOH of CDCA to 7-βOH, in which the difference-direction isomerization is completed. Microorganisms such as Bacteroides, Eubacterium, Clostridium, and Ruminococcus can oxidize and isomerize BAs.^[18]^

### 3.4. Recombination

**Figure 2.**
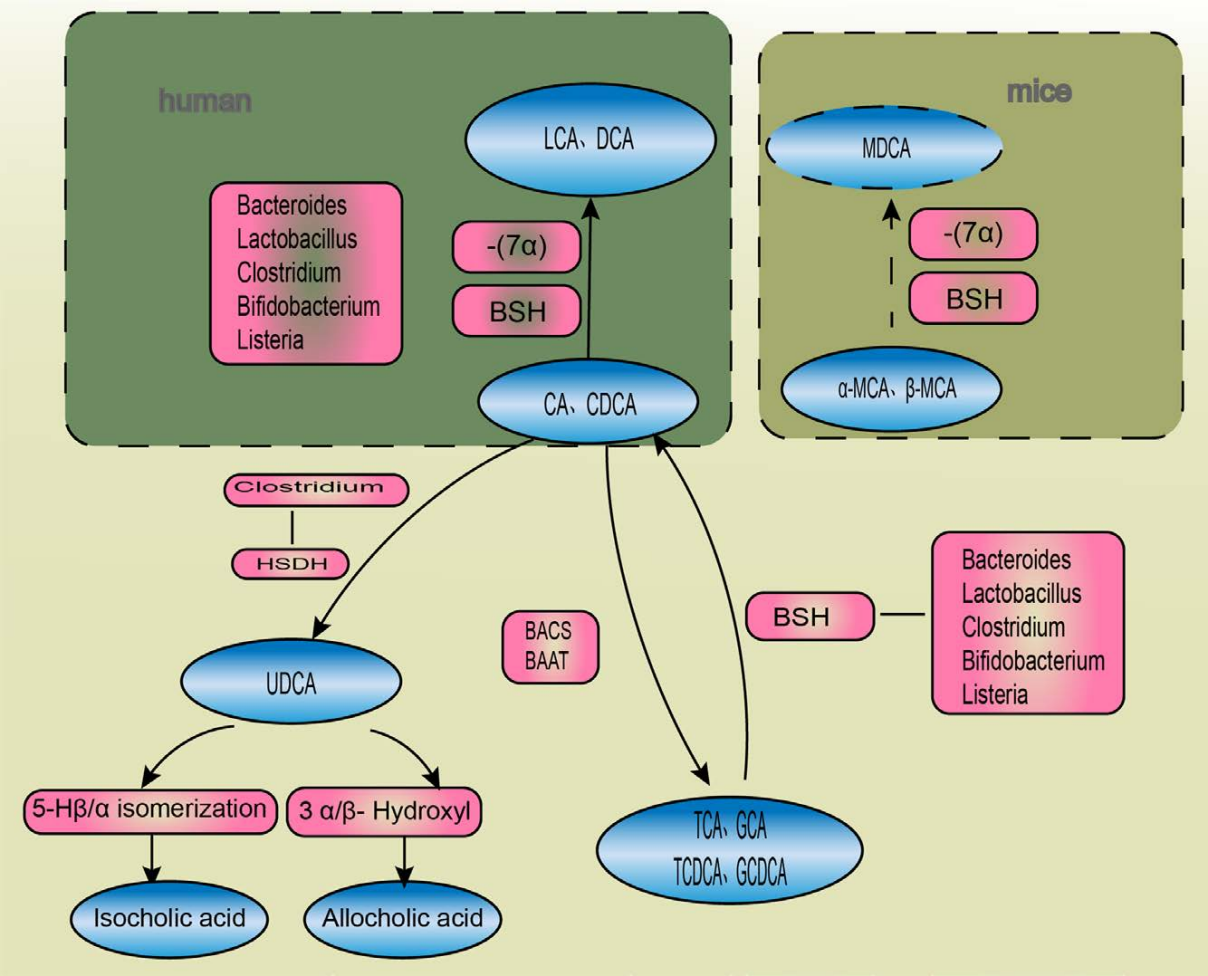
After releasing hepatocyte-derived conjugated bile acids (BAs) into the intestine, bile salt hydrolase (BSH) produced by gut microbiota hydrolyzes them into free BAs. Cholic acid (CA) and chenodeoxycholic acid (CDCA) can be converted to lithocholic acid (LCA) and deoxycholic acid (DCA) after adding 7-dehydroxyl group by BSH. Ursodeoxycholic acid (UDCA) can also be generated by the hydroxyl oxidation of 3, 7, or 12 rings by hydroxysteroid dehydrogenase (HSDH). UDCA can be converted into isocholic acid and allocholic acid by 3α/β-hydroxyl and 5-Hβ/α isomerization, respectively. Murine α-MCA and β-MCA undergo the same reaction to form MDCA.

In addition to the aforementioned pathways, recombination is a transformation method found in recent years. Similar to hepatocytes, intestinal flora can rebind free BAs. Instead of traditional taurine and glycine, Enterocloster bolteae can bind phenylalanine, leucine, and tyrosine to the carboxyl position of C24. These new BAs are called "microbe-conjugated bile acids"(MCBA), suggesting that gut microbiota uses additional mechanisms to prepare BAs for excavation.^[19]^ Figure 2 displays the relationship between gut microbiota and BA metabolism.

## 4. HF and BAs

BAs are synthesized by gut microbiota. They are crucial for regulating lipid and energy metabolism, nutrient absorption, and preserving gut homeostasis.^[20]^ Emerging evidence indicates that BAs can possibly control cardiovascular function. BAs interact with farnesoid (FXR) and pregnane/steroid and xenobiotic receptors, 2 nuclear receptors, in the heart and systemic circulation.^[21]^ BAs can reduce heart rate by controlling calcium channel conductance in sino-atrial and ventricular cardiomyocytes. They also control vascular tonicity through both endothelium-dependent and independent mechanisms.^[22]^

In individuals with HF, decreased cardiac output reduces hepatic arterial blood flow and liver congestion, which can affect liver function and BAs excretion, compromise gut and hepatic circulation, and increase BAs levels. Recently, Mayerhofer and colleagues showed that the secondary-to-primary BAs ratio is elevated in patients with HF.^[21]^ It indicates that BAs metabolism, rather than BAs level, is involved in chronic HF. On the other hand, it was recently discovered that high levels of total serum BAs can contribute to liver-associated cardiac dysfunction in the mice model, possibly through impaired oxidation of cardiac fatty acids. These findings suggest that the BAs pool, rather than specific BAs, is involved in HF.^[23]^

### 4.1. BAs production

According to their structural differences, BAs can be divided into 2 groups: free BAs, such as CA, DCA, CDCA, and LCA; and conjugated BAs, such as Glycocholic acid, Glycodeoxycholic acid, and taurocholic acid. Taurocholic acid and Glycocholic acid are produced by the combination of taurine or glycine and BAs. Primary BAs, such as CA and CDCA, are directly produced from cholesterol in hepatocytes. DCA and LCA are secondary BAs produced by dehydroxylation. Primary BAs are processed by gut microorganisms.^[24]^

#### 4.1.1. Gut microbiota synthesis of BAs.

Primary BAs can be produced through 2 pathways: the classical pathway and the alternative pathway. The majority of BAs are synthesized via the classical pathway. Cholesterol-7α-hydroxylase (CYP7A1) is the first enzyme in the classical pathway, and then steroid-12α-hydroxylase (CYP8B1) mediates 12α-hydroxylation. Thereafter, cholesterol-27-hydroxylase (CYP27A1) catalyzes 27α-hydroxylation. Then, side chain breakage and other steps occur to finally generate CA and CDCA. A small proportion of BAs are produced through the alternative pathway, in which CYP27A1 catalyzes cholesterol production, and cholesterol oxysterol 7α-hydroxylase (CYP7B1) generates CDCA as a primary BA.^[25]^

#### 4.1.2. The enterohepatic circulation of BAs.

After being synthesized in the liver, BAs are stored in the gallbladder. Eating stimulates BAs secretion into the intestine, and 95% of BAs are reabsorbed, whereas the remaining is expelled with feces. Through active and passive transport mechanisms, the conjugated and free BAs are reabsorbed, respectively. The portal vein carries the reabsorbed BAs, including primary and secondary BAs and conjugated and free BAs, into the liver. The liver converts free BAs into conjugated BAs, which are then released with bile into the intestinal lumen, "Enterohepatic circulation of BAs" is the name of this process. Reusing the scarce BAs and fostering the digestion and absorption of lipids are the physiological purposes of the enterohepatic circulation of BAs.

**Figure 3.**
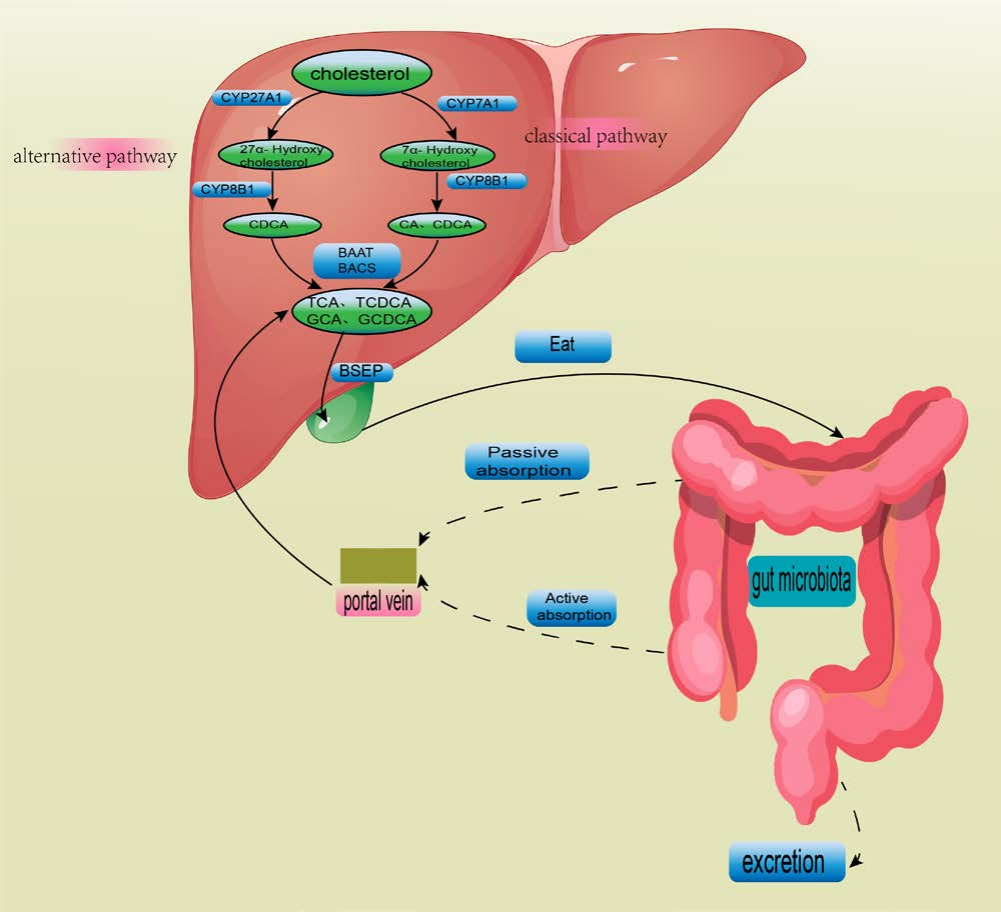
In the liver, cholesterol is a substrate for CYP7A1. It undergoes CYP8B1-mediated 12α-hydroxylation and CYP27A1-mediated 27α-hydroxylation to generate cholic acid (CA) and chenodeoxycholic acid (CDCA). A small proportion of BA production relies on the alternative pathway and CYP27A1, which converts cholesterol into 27α-hydroxycholesterol. Then, CYP7B1 converts 27α-hydroxycholesterol into CDCA. Bile acids (BAs) are combined with glycine or taurine to form conjugate BAs. Conjugated BAs lose glycine or taurine to produce free BAs. BAs are synthesized in the liver and accumulated in the gallbladder. They are secreted into the intestine after eating, and about 95% of BAs are reabsorbed in the intestine through active and passive pathways. Reabsorbed BAs return to the liver through the portal vein, and the remaining is excreted with feces.

On average, the human liver only contains 3 to 5 grams of BAs. The liver has to generate 16 to 32 grams of lipids every day to maintain lipid digestion and absorption. Inadequate production of BAs can be compensated by the enterohepatic circulation of BAs. It maximizes the emulsification of the restricted BAs pool and supports regular digestion and absorption of lipids. The enterohepatic circulation can be repeated 2 to 4 times after each meal. BAs cannot be recycled when the enterohepatic circulation is impaired; for instance, in cases of partial ileectomy or diarrhea. Impairment of enterohepatic circulation increases saturated biliary cholesterol levels, thereby enhancing the risk of cholesterol stones. On the other hand, it influences the digestion and absorption of lipids.^[25]^ Figure 3 displays BA metabolism and enterohepatic circulation.

### 4.2. The function of BAs

#### 4.2.1. Enhancing the digestion and absorption of lipids.

BAs have hydrophobic methyl, hydrocarbon nuclei, and hydrophilic hydroxyl and carboxyl groups. The spatial coordination of hydroxyl and carboxyl groups all exist at the same time. Therefore, BAs primary structure contains hydrophilic and hydrophobic groups, providing interfaceal activity. BAs can lower the surface tension between oil and water and enhance lipid emulsification. They can help the absorption and digestion of medications, dietary fat, cholesterol, and fat-soluble vitamins.^[26]^

#### 4.2.2. Prevention of gallstone formation.

Cholesterol cannot be dissolved in water. Cholesterol easily precipitates in the gallbladder due to oversaturation. However, bile contains BA salts and lecithin, which help micelle formation and prevent gallstone formation.^[27]^

#### 4.2.3. BAs as signaling molecules.

BAs also function as signaling molecules. They activate the nuclear receptor FXR and the cell surface receptor G-protein coupled receptor 5 (TGR5). BAs regulate homeostatic pathways, glucose, triglyceride, and cholesterol energy metabolism, inflammation, tumorigenesis, and cell proliferation. In order to prevent excessive BAs production under physiological or pathological situations, it is crucial to maintain the proper balance between BAs species and quantities.^[28]^

### 4.3. The involvement of BAs in HF

#### 4.3.1. Anti-inflammatory and anti-oxidant properties.

Inflammation and HF are connected. Patients with HF have higher levels of inflammatory mediators. Inflammation significantly affects the incidence, progression, and prognosis of HF. Numerous experimental studies have demonstrated that proinflammatory cytokines such as interleukin-1 (IL-1), interleukin-6, interleukin-8, and tumor necrosis factor-α (TNF-α) have higher levels in individuals with HF. Patients with HF have progressive congestion that is linked to the increased levels of inflammatory mediators,^[29,30]^ such as IL-1, interleukin-8, interleukin-6, and TNF-α.^[31]^

Recently, it has been revealed that gut microbiota is important for both maintaining health and causing diseases. By triggering the inflammatory pathways, gut dysbiosis helps the development of HF. Therefore, the potential association between gut dysbiosis and HF is worth investigating.^[32]^

FXR is a nuclear hormone receptor known to play a key role in the metabolism of BAs. It is strongly activated by CDCA followed by DCA, CA, and LCA. The tissue-specific expression of FXR alters the mechanistic roles they play in maintaining BA homeostasis.^[33]^ Modest concentrations of CDCA can interact with FXR to attenuate inflammation in vascular smooth muscle and prevent coronary atherosclerosis.^[34]^ BAs can also prevent cardiac remodeling and hypertrophy by inhibiting nuclear factor-κB (NFκ-B), a transcription factor for several inflammatory mediators.^[35]^

Evidence from clinical and experimental studies has shown that HF increases systemic and myocardial oxidative stress.^[36–38]^ By controlling redox pathway, BAs can protect the myocardium, both TCA and TCDCA have been shown to increase the expression of peroxisome proliferator-activated receptor gamma, Ras-related C3 botulinum toxin substrate 1, NADPH oxidase 4, *p21CIP*, and endothelial nitric oxide synthase.^[39]^ Additionally, TGR5 overexpression reduced oxidative stress and inflammation by activating the AKT pathway in the cardiac myoblast cell line. LCA inhibited high glucose-induced cardiac hypertrophy in H9c2 cells,^[40]^ in addition, TGR5 activation enhanced myocardial survival.^[41]^

#### 4.3.2. Protective effects of BAs on mitochondrial dysfunction.

Mitochondria are surrounded by 2 membranes to provide energy for normal cellular function. As it consumes too much energy, the heart must constantly produce ATP to support muscle contraction and relaxation. In normal myocardial mitochondria, fatty acids are used as the principal fuel for β-oxidation, which produces about 70% of myocardial ATP. Fatty acids can even provide up to virtually 100% of the total myocardial energy. With the development of HF, myocardial fuel gradually shifts from fatty acids to glucose.^[42]^

In addition, mitochondria-mediated cardiomyocyte apoptosis plays a significant role in the development of HF. Impaired mitochondrial structure and function can lead to cell apoptosis, myocardial metabolic remodeling, and chronic HF.^[43]^ Therefore, improving mitochondrial function is pivotal for treating HF.

Previous studies have increasingly shown that end-stage HF is the result of mitochondrial dysfunction. Additionally, recent studies have shown that mitochondrial dysfunction has a role in oxidative stress and cardiomyocyte death, forming a vicious cycle in the failing heart.^[44]^

In addition, secondary BAs can affect the function of the intestinal barrier, inflammation, and mitochondrial biosynthesis.^[45]^ Some scientists reported that gut microbiota may indirectly upregulate SIRT1 and FXR via BAs to control mitochondrial function.^[46]^

#### 4.3.3. Inhibiting cardiomyocyte apoptosis.

Cardiomyocyte apoptosis plays a key role in the development of early HF. Extensive cardiomyocyte apoptosis can deteriorate myocardial function in HF. Inadequate blood supply and increased oxygen consumption accelerate cardiomyocyte death and exacerbate ventricular remodeling in HF. Therefore, improved cardiac energy metabolism can decrease cardiomyocyte death. Consistently, cardiomyocyte apoptosis has been linked to HF in a mice model.^[47]^

The plasma level of BAs can affect the cardiovascular system in both directions. High levels of BAs can stimulate FXR nuclear receptor and activate mitochondrial death signal and apoptosis.^[48]^ The experimental studies uncovered that UDCA can treat prenatal cardiomyocyte dysfunction.^[49]^

### 4.4. Signaling pathways of BAs in treat HF

#### 4.4.1. FXR signaling pathway.

FXR is a member of a nuclear receptor superfamily that is crucial for controlling inflammatory response, oxidative stress, and glucose and lipid metabolism. Recently, it has been demonstrated that FXR is expressed in adult cardiomyocytes, myocardial tissue, and vascular wall. Numerous investigations have indicated the function of FXR in the cardiovascular system. FXR controls BAs production, cholesterol metabolism, inflammation, oxidative stress, cell death, and vascular remodeling.^[48]^

Pu et al showed that FXR is expressed in cardiac cells and its activation significantly induces apoptosis through mitochondrial death signaling. They showed that FXR signaling is implicated in several cardiac diseases. They further confirmed their findings in an animal model of myocardial ischemia/reperfusion injury.^[48]^ By triggering the AMPK signaling pathway, FXR ameliorates ET-1-mediated cardiomyocyte injury.^[50]^ All of these findings indicate that the FXR receptor may play a role in the development and progression of HF by influencing lipid and glucose metabolism, oxidative stress, inflammation, cell death, and vascular remodeling.

FXR is a nuclear receptor that is activated by BAs. After activation, FXR controls important genes that are involved in the production, transportation, and reabsorption of BAs, and in the metabolism of carbohydrates and lipids.^[51]^ The most potent ligand of FXR is CDCA, followed by CA, DCA, and LCA. UDCA does not activate FXR. It rather inhibits FXR activation.^[52]^ These findings suggest that after FXR activation, BAs can contribute to the development of HF by modulating several signaling pathways.

#### 4.4.2. Vitamin D receptor (VDR) signaling pathway.

The vitamin D receptor (VDR) is a nuclear receptor for steroid hormones with a significant impact on gene transcription. VDR is a gene regulator that may be particularly relevant to cardiovascular disease and HF.^[53]^ Numerous targets of the VDR are related to cardiac diseases. Previous findings suggest that changes in the vitamin D axis are linked to cardiac diseases. In several animal models, it has been demonstrated that vitamin D prevents ventricular hypertrophy and cardiac dysfunction.^[54]^

ANP and BNP are biomarkers of HF since they are components of the RAS counter-regulatory mechanism. Chen and colleagues showed that isoproterenol-induced ventricular hypertrophy activates VDR expression and hBNP promoter, suggesting a direct relationship between VDR and hBNP promotor.^[55]^ Previous studies revealed that VDR is a more sensitive BA receptor than FXR and pregnane X receptor (PXR), particularly for LCA and its main metabolite 3-keto-LCA55. It was also uncovered that VDR may be activated by CA, a secondary BA stones.

#### 4.4.3. Pregnane X receptor (PXR) signaling pathway.

PXR is a nuclear receptor activated by various chemical stimuli, including gut microbial metabolites. It plays a crucial role in controlling the inflammatory response and maintaining the function of intestinal epithelium under both normal and inflammatory conditions. Vascular endothelium and smooth muscle cells both express PXR.

In vascular endothelial cells, PXR can increase the activity of phase I and phase II oxidases, transporters, and enzymes associated with oxidative stress. It can also mitigate endothelial dysfunction caused by H_2_O_2_.^[56]^ PXR activation in endothelial cells controls toll-like receptors (TLR) and nucleotide-binding oligomerization domain-like receptors to maintain immune/inflammatory balance. PXR activation can directly upregulate TLR2, TLR4, TLR9, nucleotide-binding oligomerization domain-containing protein 1, and nucleotide-binding oligomerization domain-like receptor family pyrin domain protein 3 (NLRP3), thereby leading to caspase-1 and IL-1 shear maturation.^[57]^

Activated PXR can control gene expression and contribute to the treatment of cholestatic liver disease.^[58]^ PXR may play a role in the incidence and progression of HF based on its function in immune response, oxidative stress, and inflammation.

#### 4.4.4. TGR5 signaling pathway.

In 2003, TGR5 was discovered by Kawamata using the human spleen cDNA library for GPCR after being first reported by Japanese researchers in 2002.^[59,60]^ TGR5 is a G-protein-coupled receptor (GPCR) that is expressed in various tissues, including adipose tissue, muscular tissue, immune cells, spinal cord, and enteric nervous system. TGR5 inhibits atheroma formation and myocardial inflammation. In bovine aortic endothelial cells, activated TGR5 promoted nitric oxide production and inhibited NF-κB activity, thereby suppressing monocyte adhesion and preventing the development of atherosclerosis.^[61]^

**Figure 4.**
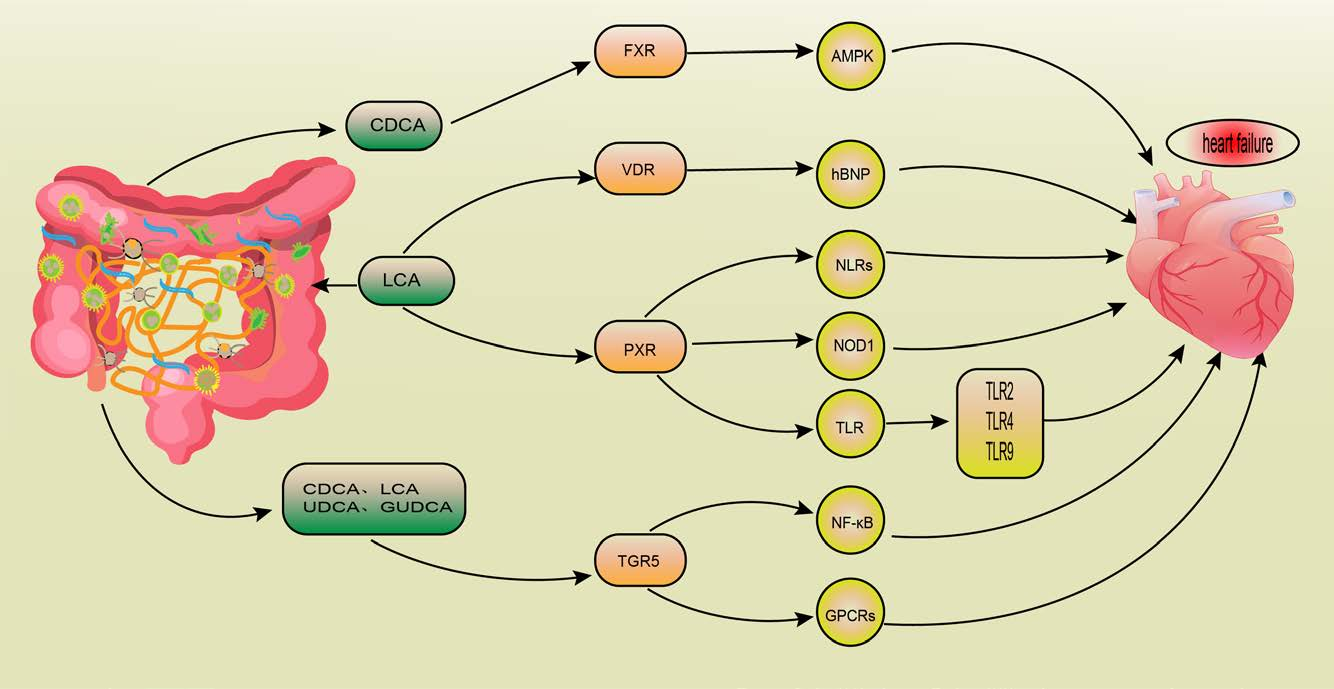
Bile acids (BAs) mainly affect heart failure (HF) through farnesoid (FXR), vitamin D receptor (VDR), pregnane X receptor (PXR), and G-protein coupled receptor 5 (TGR5) receptors. The most effective ligand for FXR is chenodeoxycholic acid (CDCA) and VDR. PXR can be activated by lithocholic acid (LCA). CDCA, ursodeoxycholic acid (UDCA), LCA, and GUDCA can activate TGR5.

BAs, especially TGR5 agonists, increased myocardial response to inotropes, and hemodynamic stress in mice. TGR5 may be a potential therapeutic target for HF since it plays a significant role in myocardial adaptation.^[41]^ Figure 4 displays that BAs regulate several signaling pathways in HF.

## 5. Traditional Chinese medicine (TCM) treats HF by regulating gut microbial metabolites and BAs

TCM has a long history of treating HF with extensive experience. TCM has a thorough understanding of the location, etiology, pathophysiology, and process of HF. It uses decoction, Chinese herbal compounds, Chinese herbal formulas, Chinese patent medicine, acupuncture, and other treatment methods to treat diseases, with fewer side effects, and acceptable efficacy. It also has a holistic approach and emphasizes syndrome differentiation and treatment as the treatment idea. TCM successfully alleviates immune response and improves objective measures such as left ventricular ejection fraction and N-terminal pro-brain natriuretic peptide.^[62]^

TCM can protect the intestinal mucosal barrier, restore gut microbial diversity, and enhance immune function by regulating the number and proportion of intestinal microflora, inflammatory factors, signaling pathways, and genes. There are many similarities between intestinal microecology and Chinese medicine theory, such as the holistic concept and the theory of the “heart is connected with the small intestine.” These similarities provide a theoretical basis to prevent and treat diseases by regulating intestinal microecology, suggesting that the cross-talk between gut microbiota and the heart may become a new therapeutic target for HF.^[63]^ Several herbal medications can treat diseases by altering gut microbial composition and its metabolites. These medications are frequently used in the management of HF.^[63]^ TCM-based modulation of gut microbiota has received much attention for preventing HF.^[64]^ Additionally, TCM has been shown to control BAs production and BA transporter gene expression and regulate pathways related to BA transportation.^[65]^

### 5.1. Chinese herbal compounds

The active components of traditional Chinese medications can trigger the necessary signal pathways. Due to their function as FXR and TGR5 agonists, coptis alkaloids, the active component of traditional Chinese medications, have lipid-lowering properties.^[66]^ In recent years, 130 different components of TCM have been screened for PXR activation. Several compounds with potential CYP3A4 induction and inhibition ability have been identified, including artemisinin, glycyrrhizic acid, and tanshinone IIA.^[67]^ Wu^[68]^ et al found that quercetin can affect primary BA biosynthesis by regulating gut microbiota, thereby reducing TC, TG, HDL, LDL, TNF-α, and IL levels and improving vascular damage.

### 5.2. Chinese herbal formulas

Numerous studies have demonstrated that TCM can affect the composition and function of gut flora, and improve BAs production. Xiaoqinglongtang regulated gut microbial composition, ameliorated myocardial fibrosis, cardiac hypertrophy, and inflammatory cell infiltration, and decelerated HF progression in hypertensive rats.^[69]^ Si-Miao-Yong-An Tang considerably increased the production of taurocholic acid, and glycine CA, and significantly lowered cholesterol levels in a mice model of hyperlipidemia. Simiaoyong decoction may cause a significant decrease in cholesterol level by promoting the conversion of cholesterol into BAs.^[70]^ Chaihu Shugansan dramatically increased TUDCA production in rats with slight liver damage.^[71]^ Xiexin Decoction decreased intimal damage in a mice model of atherosclerosis. It also improved gut dysbiosis, promoted BA synthesis, increased BA pool, and reduced proinflammatory factors and lipid levels.^[72]^ Yinchenzhufutang effectively improved abnormal BAs homeostasis, BAs transporter, inflammatory cell infiltration and other pathological damage.^[73]^

### 5.3. Chinese patent medicine

**Figure 5.**
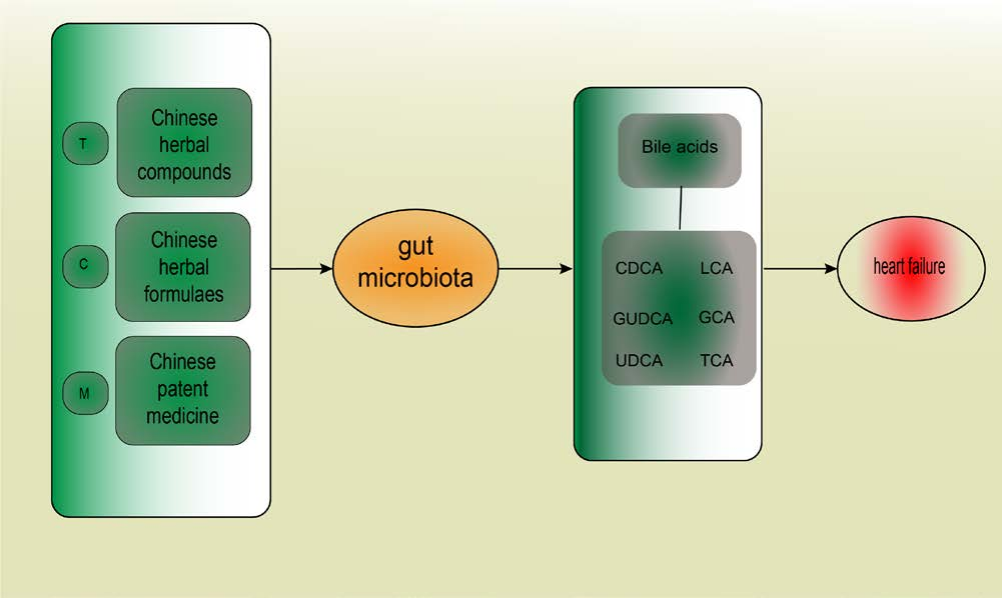
Chinese herbal compounds, Chinese herbal formulas, and Chinese patent medicine can treat heart failure (HF) by modulating gut microbiota, thereby improving bile acids (BAs) levels and activating the related pathways.

Qishen Granule is frequently used to treat and alleviate myocardial ischemia and HF. Qishen Granule can regulate the production of UDCA, glycodeoxycholic acid, and other BAs in the rat model of HF.^[74]^ Figure 5 displays the role of Chinese medicine in treating HF by regulating BAs.

## 6. Conclusions and perspective

Gut microbiota and HF have interwoven relationships. Gut microbiota produces BAs, a bile metabolite that is crucial for lipid absorption, regulation of energy metabolism, and preserving intestinal homeostasis. BAs may regulate cardiovascular function by modulating cardiac inflammation, cardiomyocyte apoptosis, oxidative stress, mitochondrial dysfunction, and other processes via receptors, such as FXR, VDR, PXR, TGR5, and muscarinic receptor, and channels, such as BK channel.

TCM offers extensive experience in treating HF by focusing on gut microbiota. TCM has paid more attention to gut flora and BA in cardiovascular diseases. Chinese herbal medicine like Coptis chinensis, Artemisia annua, Glycyrrhiza uralensis, Salvia miltiorrhiza, Bupleurum chinense, Chaihu Shugan Powder, Simiao Yong'an Decoction, Xiexin Decoction, Chinese patent medicine Qishen granules, and herbal cake separated moxibustion reduce cardiomyocyte apoptosis and oxidative stress by improving gut dysbiosis. These findings indicate that TCM can treat HF by controlling gut flora and BAs. How TCM controls gut flora and uses BA to treat HF, remains to be known. Although FXR, VDR, PXR, TGR5, and muscarinic receptors are expressed in the cardiovascular system, there is only indirect evidence that BAs interact with these receptors to improve HF. It is therefore necessary to examine the mechanism of Chinese medicine in treating HF by focusing on gut microbiota and BAs.

## Author contributions

**Conceptualization:** Min Shi, Zhihua Guo.

**Formal analysis:** Min Shi, Jiaming Wei.

**Funding acquisition:** Ya Li, Zhihua Guo.

**Supervision:** Zhihua Guo.

**Validation:** Ya Li, Zhihua Guo.

**Writing – original draft:** Min Shi, Hui Yuan.

**Writing – review & editing:** Min Shi, Zhihua Guo.
